# Making Sense of a Scent-Sensing Metaphor for Microbes and Environmental Predictions

**DOI:** 10.1128/mSystems.00993-21

**Published:** 2021-08-31

**Authors:** Jeff S. Bowman

**Affiliations:** a Scripps Institution of Oceanography, UC San Diego, La Jolla, California, USA; b Center for Microbiome Innovation, UC San Diego, La Jolla, California, USA

**Keywords:** 16S rRNA gene, machine learning, microbial community structure, random forest

## Abstract

Microbes serve as sensitive indicators of ecosystem change due to their vast diversity and tendency to change in abundance in response to environmental conditions. Although we most frequently observe these changes to study the microbial community itself, it is increasingly common to use them to understand the surrounding environment. In this way microbial communities can be thought of as powerful sensors capable of reporting shifts in chemical or physical conditions with high fidelity. In this commentary, I further explore this idea by drawing a comparison to the olfactory system, where populations of sensory neurons respond to the presence of specific odorants. The possible combinations of sensory neurons that can transduce a signal are virtually limitless. Yet, the brain can deconvolute the signal into recognizable and actionable data. The further development of machine learning techniques and its application hold great promise for our ability to interpret microbes to detect environmental change.

## COMMENTARY

There is urgent need to understand environmental change in our complex and dynamic world. The ability to predict important but difficult-to-measure environmental parameters can help us detect change and evaluate potential causation. Here, I describe how microbial communities may be used as highly sensitive indicators of ecosystem change, analogous to the way that olfactory sensory neurons respond to environmental stimuli to produce smell. The olfactory system contains millions of individual sensory neurons. We can think of these neurons as being divided into populations, with each population capable of sensing a range of chemically related odorants based on which odorant receptors they contain (1). The concept of smell is an emergent property of this system; the signal pattern from a given collection of sensory neurons is recognized by the brain as corresponding to a specific smell. Like sensory neurons, a microbial community is composed of populations of distinct members that respond to environmental stimuli. However, unlike neurons these microbes do not respond via an electrical impulse. Rather, they increase or decrease as a proportion of the total population based on the impact of the stimulus on their fitness relative to the other members of the community. It is possible to reconstruct the ecosystem state or even the intensity of the specific stimuli that induced these changes if we can interpret them with sufficient accuracy.

**FIG 1:**
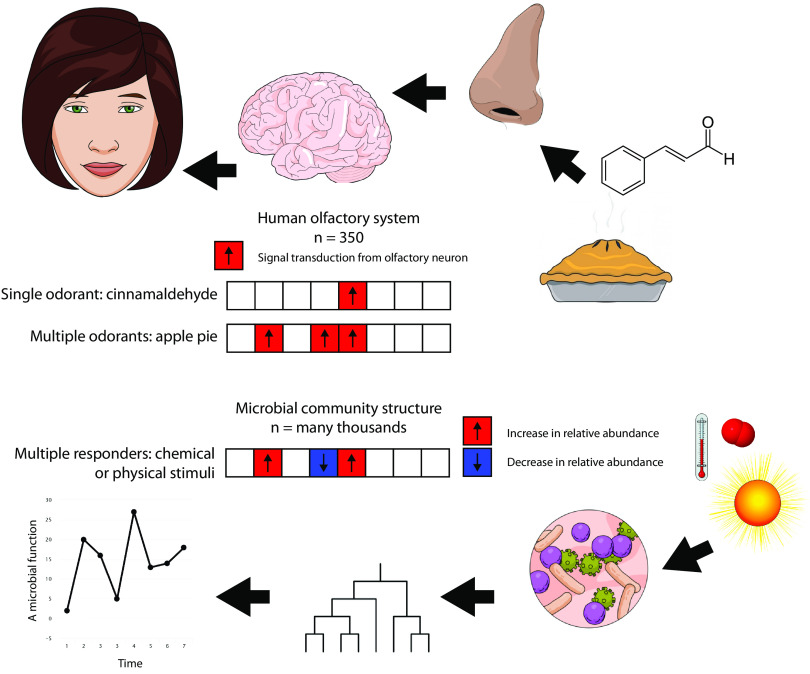
Environmental sensing with the olfactory system and microbial community structure. The human olfactory system contains roughly 350 types of olfactory sensory neurons. A single odorant (such as cinnamaldehyde) will activate a single type of sensory neuron. Many smells are the synthesis of multiple odorants; the combination of olfactory sensory neurons transducing a signal must be decoded by the brain to achieve a specific sensory response. Populations of microbes respond to environmental stimuli (such as temperature, sunlight, or O_2_) by increasing or decreasing relative to other members of the community. Machine learning techniques such as random forest regression can be used to decode these signals, providing a prediction of specific parameters for which an adequate model exists.

In the abstract, the brain’s interpretation of a combination of incoming signals as a specific smell is an example of classification (Fig. 1). The signals from many different neurons—varying in intensity based on the affinity of the receptor to the odorant—are combined into a singular solution (e.g., apple pie, the sea, wood smoke) that we might term a categorical variable. Classification is already widely used to relate microbial community structure to categorical variables that describe ecosystem state, particularly where the ecosystem is the human body, and a supervised machine learning approach is appropriate. Examples include work by Duvallet et al. (2), who used random forest classification to relate community structure to disease state, and Pasolli et al. (3), who tested a number of classification algorithms including random forest, support vector machines, and lasso and elastic net classification to predict disease state. But what if we wish to predict a continuous variable rather than a categorical one? Returning to the olfactory metaphor, what if we could interpret the output of olfactory sensory neurons with sufficient fidelity to determine the concentration of specific odorants in the environment?

Early evidence suggests that the structure of a microbial community contains sufficient information to reproduce key attributes of the environment that are continuous variables. Bowman et al. (4) applied an unsupervised self-organizing map (SOM) approach to “segment” a marine microbial community into different classes, showing that these classes could be used to predict (continuous) ecophysiology traits in a generalized linear modeling framework. Belk et al. (5) used random forest regression to predict cadaver postmortem interval from microbial community structure, and Thompson et al. (6) used a similar approach to predict the concentration of dissolved organic carbon (DOC) in soil microcosms. In recent work, Dutta et al. (A. Dutta, T. Goldman, J. Keating, E. Burke, N. Williamson, D. Reinhard, and J. S. Bowman, submitted for publication) used random forest regression to predict hydrogen sulfide concentration in a microcosm model of a souring oil production field. Constructing a model from over 400 samples, those authors were able to determine hydrogen sulfide concentration in 174 samples (withheld from the training data) with surprising accuracy (*R*^2^ = 0.83). The outstanding question is, how many ecological parameters are sufficiently captured by microbial community structure to be predicted in this way?

The prediction of ecosystem attributes from microbial community structure works because microbes exist in a close causal relationship with the physical and chemical environment. This is easily imagined for environmental chemistry where, for example, specific microbes will grow preferentially in the presence of a substrate or signaling molecule and produce metabolic by-products and signaling molecules in turn. Given enough parallel observations of both community structure (e.g., with 16S, 18S, and/or internal transcribed spacer [ITS] rRNA gene sequencing) and a chemical compound of interest that interacts with that community, it will be possible to predict the concentration of the compound from the structure of the community. The microbial community has a similarly close relationship with physics. Changes in temperature, salinity, water activity, and solar irradiance can induce a rapid shift in community structure, while the microbial community influences such physical environmental parameters as soil porosity and light transmittance.

A key requirement for predicting environmental parameters from microbial community structure is that enough well-constrained observational data exist to train a model. These data need to include both microbial community structure (the predictor variables) and either a continuous or categorical response variable. As with all models, to achieve good performance the observational data must fit the range of environmental conditions over which predictions are desired. Microbial community structure data typically consist of hundreds to thousands of predictor variables as unique sequence reads when adequately quality controlled (QC’d) and denoised. The breadth of these data presents a prime opportunity for model overfitting. To reduce this risk, the number of samples used to train the model should be large, or the number of features used in the final model should be down-selected to the minimum number required to produce a model of sufficient performance. This is not without risk, however; feature down-selection may increase the specificity of the model while minimizing overtraining for the limited range of conditions over which the model is expected to perform well. Consider a hypothetical model that predicts soil nitrogen fixation from microbial community structure. A model based only on those features deemed most informative for, e.g., a soybean field is not likely to produce good predictions for a wheat field. Because functional potential is thought to be more conserved than taxonomic structure across microbial communities occupying similar environments (7, 8), models based on functional genes may be the most broadly applicable though less sensitive than models based on taxonomic marker genes such as the 16S rRNA gene.

It is important to recognize that while the microbial community may accurately represent some environmental parameters, the inverse is not true. Microbial community structure may yield a unique solution for many environmental parameters, but there is no reason to expect that a given set of environmental parameters could be used to predict a specific microbial community, just as we could not predict the exact pattern of activated sensory neurons yielding a specific smell in our olfactory metaphor. Even the combination of compounds that yields a specific smell is not unique; the assemblages of odorants in, e.g., apple pie and apple-pie-scented potpourri are not the same but yield similar scents. The microbial scenario is consistent with our current understanding of the impact of disturbance on microbial community structure. Although ecosystem function may be recovered on the return to baseline conditions following disturbance, the taxonomic structure of the community is likely to be different (9, 10). Thus, similar niches can host very different microbial communities.

The tight coupling between microbes and their environment provides an opportunity to use microbiomes as sensitive indicators of ecosystem change. The power of this opportunity lies in the vast diversity of microbial communities, which contain many thousands of genotypes within even a liter of seawater or a few grams of soil. Each of these taxa responds uniquely to many different chemical or physical stimuli, expressing their change in fitness within the community as a shift in relative abundance. Even a highly simplified microbial community of 100 taxa—and considering only upshift, downshift, or no shift in relative abundance—can yield 3^100^ distinct signals. The challenge is specificity and our ability to interpret the signal output from this environmental sensor. Advances in analytical techniques, particularly machine learning algorithms such as random forest, provide an opportunity to develop predictive models of key environmental parameters from microbial community structure data. To take advantage of this opportunity, we must emphasize bold, high-resolution observational data sets that span key ecological gradients and measure broad suites of relevant parameters over space and time.

Approximately 350 distinct classes of sensory neurons are present within the human olfactory system, far less diversity than is present in most microbial communities. Simplified to a binary response (i.e., ignoring signal intensity), this system would still be capable of 2^350^ distinct signals. As reviewed by Cleland (1), the brain has evolved a complex, multilayered computing structure to address the complexity inherent in this system. It is no accident that neural networks—a cornerstone of machine learning—are modeled on the architecture of the human brain. Our ability to deconstruct complex sensory inputs into interpretable and actionable data about our environment holds great promise for our ability to use microbial community structure to understand environmental change.

## References

[1] Cleland TA. 2014. Construction of odor representations by olfactory bulb microcircuits. Prog Brain Res 208:177–203. doi:10.1016/B978-0-444-63350-7.00007-3. 24767483

[2] Duvallet C, Gibbons SM, Gurry T, Irizarry RA, Alm EJ. 2017. Meta-analysis of gut microbiome studies identifies disease-specific and shared responses. Nat Commun 8:1784. doi:10.1038/s41467-017-01973-8. 29209090 PMC5716994

[3] Pasolli E, Truong DT, Malik F, Waldron L, Segata N. 2016. Machine learning meta-analysis of large metagenomic datasets: tools and biological insights. PLoS Comput Biol 12:e1004977. doi:10.1371/journal.pcbi.1004977. 27400279 PMC4939962

[4] Bowman JS, Amaral-Zettler LA, Rich JJ, Luria CM, Ducklow HW. 2017. Bacterial community segmentation facilitates the prediction of ecosystem function along the coast of the western Antarctic Peninsula. ISME J 11:1460–1471. doi:10.1038/ismej.2016.204. 28106879 PMC5437343

[5] Belk A, Xu ZZ, Carter DO, Lynne A, Bucheli S, Knight R, Metcalf JL. 2018. Microbiome data accurately predicts the postmortem interval using random forest regression models. Genes (Basel) 9:104. doi:10.3390/genes9020104. PMC5852600 29462950

[6] Thompson J, Johansen R, Dunbar J, Munsky B. 2019. Machine learning to predict microbial community functions: an analysis of dissolved organic carbon from litter decomposition. PLoS One 14:e0215502. doi:10.1371/journal.pone.0215502. 31260460 PMC6602172

[7] Bowman J, Ducklow H. 2015. Microbial communities can be described by metabolic structure: a general framework and application to a seasonally variable, depth-stratified microbial community from the coastal West Antarctic Peninsula. PLoS One 10:e0135868. doi:10.1371/journal.pone.0135868. 26285202 PMC4540456

[8] Louca S, Jacques SMS, Pires APF, Leal JS, Srivastava DS, Parfrey LW, Farjalla VF, Doebeli M. 2016. High taxonomic variability despite stable functional structure across microbial communities. Nat Ecol Evol 1:15. doi:10.1038/s41559-016-0015. 28812567

[9] Santillan E, Constancias F, Wuertz S. 2020. Press disturbance alters community structure and assembly mechanisms of bacterial taxa and functional genes in mesocosm-scale bioreactors. mSystems 5:e00471-20. doi:10.1128/mSystems.00471-20. 32843539 PMC7449608

[10] David LA, Materna AC, Friedman J, Campos-Baptista MI, Blackburn MC, Perrotta A, Erdman SE, Alm EJ. 2014. Host lifestyle affects human microbiota on daily timescales. Genome Biol 15:R89. doi:10.1186/gb-2014-15-7-r89. 25146375 PMC4405912

